# The Modulating Effect of Ursodeoxycholic Acid on Liver Tissue Cyclooxygenase-2 Expression Following Extended Hepatectomy

**DOI:** 10.7759/cureus.15500

**Published:** 2021-06-07

**Authors:** Dimitrios Papakonstantinou, Anna Paspala, Emmanouil Pikoulis, Despoina N Perrea, Anastasios Machairas, Georgios Agrogiannis, Nikolaos Machairas, Paulos Patapis, Nikolaos J Zavras

**Affiliations:** 1 Third Department of Surgery, "Attikon" University General Hospital/National and Kapodistrian University of Athens, School of Medicine, Athens, GRC; 2 Third Department of Surgery, "Attikon" University Hospital/National and Kapodistrian University of Athens, Athens, GRC; 3 Third Department of Surgery, National and Kapodistrian University of Athens, Athens, GRC; 4 Surgery, Attikon University Hospital, Athens, GRC; 5 Laboratory of Experimental Surgery and Surgical Research, National and Kapodistrian University of Athens School of Medicine, Athens, GRC; 6 First Department of Pathology, National and Kapodistrian University of Athens, School of Medicine, Athens, GRC; 7 Third Department of Surgery, "Attikon” General University Hospital/National and Kapodistrian University of Athens, School of Medicine, Athens, GRC; 8 Department of Pediatric Surgery, "Attikon" University General Hospital/National and Kapodistrian University of Athens, School of Medicine, Athens, GRC

**Keywords:** ursodeoxycholic acid, extended hepatectomy, liver regeneration, cycloxygenase-2, experimental animal study

## Abstract

Introduction: Hepatic regeneration is a complex process involving a multitude of well-timed molecular operations. Ursodeoxycholic acid (UDCA) is postulated to exert a protective effect against oxidative stress and enzymatic degradation of the extracellular matrix, in turn potentiating the regenerative response. The aim of the present animal study is to evaluate the impact of UDCA administration in liver tissue expression of cyclooxygenase-2 (COX-2) in a setting of acute liver failure achieved by 80% hepatectomy.

Materials and methods: Twenty-four adult male Sprague-Dawley rats were randomly assigned to an experimental (UDCA) and a control group. Animals in the UDCA received oral pretreatment with UDCA for 14 days via feeding tube, while animals in the control group received saline. All animals underwent resection of approximately 80% of the liver parenchyma. Tissue and blood sample collection were performed 48 hours postoperatively.

Results: The postoperative mitotic index and Ki-67 levels were found to be elevated in the UDCA group (43±11.4 and 13.7±24.7 versus 31±16.7 and 7.6±5.7), albeit without any statistical significance. Pretreatment with UDCA significantly decreased COX-2 expression levels (p=0.28) as well as serum tumor necrosis factor α (TNFα) levels (37.3±10.9 pg/mL versus 75.4±14.4 pg/mL, p=0.004). COX-2 expression score was observed to be weakly correlated to Ki-67 levels in both groups. Although COX-2 expression score was not correlated with serum TNFα levels in the control group, animals pretreated with UDCA exhibited moderate correlation (r=0.45).

Conclusion: Preoperative administration of UDCA exerts a suppressive effect on tissue expression of COX-2 following 80% hepatectomy and enforces a positive correlation between COX-2 and serum TNFα levels, suggesting that UDCA preconditions liver tissue to display an enhanced regenerative response to circulating cytokines, most notably TNFα. The weak association of COX-2 with Ki-67 expression levels suggests that COX-2 may be of secondary importance during the early phases of liver regeneration.

## Introduction

Hepatic regeneration is a complex process involving a multitude of well-timed molecular operations. In cases of liver injury, the generalized inflammatory state that accompanies the early phase of liver regeneration ushers senescent hepatic progenitor cells to enter the M phase of cell-cycle through the action of key molecular actors such as cyclin-D1 [1], NF-kB [2], and tumor necrosis factor-α (TNFα) [3]. On a local level, increased prostaglandin synthesis enhances liver regeneration, an effect that is reversible by the non-selective cyclooxygenase (COX) inhibitor indomethacin [4]. Furthermore, expression of hepatocellular COX-2 has been found to be increased in rats undergoing partial hepatectomy, while selective COX-2 inhibition predictably hampered the regenerative response [5].

In the regenerating liver, ursodeoxycholic acid (UDCA) is postulated to exert a protective effect against oxidative stress [6] and enzymatic degradation of the extracellular matrix [7], in turn potentiating the regenerative response [8]. UDCA has also been associated with decreased tissue expression of COX-2 in rodent specimens of colon cancer [9] and intestinal tissue [10], hinting at a potential immunomodulatory mechanism involving COX-2 suppression, while plausibly maintaining adequate enzymatic activity levels. The aim of this present animal study is to evaluate the impact of UDCA administration in liver tissue expression of COX-2 in a setting of acute liver failure achieved by 80% hepatectomy and further assess the immunomodulatory effects of UDCA on proliferating hepatocytes.

## Materials and methods

Animals, chemicals, and diets

Twenty-four adult male Sprague-Dawley rats were included in the present study. Based on the previous report by Paspala et al. [8], a sample size of 24 rats was deemed appropriate to detect statistical significance between the compared groups with 90% statistical power. The animals were bred by the Laboratory of Experimental Surgery “NS Christeas” of the National and Kapodistrian University of Athens according to the Directive 200/63/EU and were housed in the facilities of the same laboratory. The study protocol was approved by the competent Veterinary Directorate of Athens Prefecture, Greece (Approval No.: 908/23.02.2016). All animals included in the present study which were four months old during the time of the experiment, with a mean weight of 259.3±15.1 g, were separately housed in a controlled environment of 19 ±1 °C with 12 h light/dark cycles (light cycle from 08:00 to 20:00 h) and were fed standard laboratory chow, with feed and water provided ad libitum.

For the purposes of the experiment presented herein, the animals were randomly assigned to an intervention group (UDCA group, n=12) and a control group (n=12). No significant differences in terms of weight were observed following randomization (Table 1). The 12 rats which comprised the UDCA group received orally administered UDCA dissolved in saline via feeding tube, twice daily, at a dose of 25 mg/kg/day for 14 days. Animals in the control group received saline via feeding tube as a placebo. The UDCA solution to be infused was prepared by dissolving a 250 mg capsule (Ursofalk, Galenica SA, Athens, Greece) in 50 mL of normal saline.

**Table 1:** Baseline comparison between the intervention and control groups. *Values are expressed as median (IQR).

	Control group (n=12, mean±SD)	UDCA group (n=12, mean±SD)	p-Value
Animal weight	263.6±14.8 g	256.4±15.7 g	0.5
Mitotic Index (per 40 hpf)	31±16.7	43±11.4	0.053
Ki-67	7.6±5.7	13.7±24.7	0.073
COX-2 staining extent*	4 (1)	3 (0.5)	0.013
COX-2 staining intensity*	2.5 (2)	1 (1)	0.15
COX-2 expression score*	6 (2.5)	4 (1.5)	0.028
TNFα	75.4±14.4 pg/mL	37.3±10.9 pg/mL	0.004

Surgical procedures

Following completion of the 14-day UDCA pretreatment, the animals underwent extended hepatectomy with resection of 80% of liver parenchyma using the technique described by Martins et al. [11] which entails resection of the middle, inferior right, and left lateral lobes in order to create a setting of acute liver failure. The surgical procedures were performed under sterile conditions between 12:00 AM and 6:00 PM. Each rat was anesthetized by mask inhalation of diethyl ether and received 10 mL of intraperitoneal saline solution injection to maintain hydration. Postoperatively, each rat was individually housed in a monitored environment, receiving subcutaneous buprenorphine at a dose of 0.05 mg/kg twice daily for analgesia.

All animal procedures were performed in accordance with the European Communities Council Directive of September 22, 2010 (276/33/20.10.2010) and approved by the competent Veterinary Directorate of Athens Prefecture, Greece. The principles of 3Rs (replacement, refinement, reduction) were adhered to throughout the experiment. No adverse events were encountered during the postoperative period. The experiment was concluded 48 hours postoperatively and the rats were euthanized by exsanguination following deep sedation. Blood and liver tissue samples were collected at the end of the experiment.

ELISA and immunohistochemistry

Postoperative serum levels of TNFα were measured by enzyme-linked immunosorbent assay (ELISA) using a commercially available kit (TNFα mouse ELISA Kit, Cayman, Michigan, USA). Immunohistochemical staining for Ki-67 and COX-2 was performed on tissue slides of 5-6 μm thickness, obtained from each regenerating remnant liver specimen. Each tissue slide was deparaffinized in xylene and subsequently hydrated in ethanol solution. After rinsing with water, the tissue samples were introduced to EDTA buffer solution and left to rest for 12 minutes. Cytoplasmic COX-2 immunohistochemical staining was performed using a commercially available kit (Lyophilized Mouse Monoclonal Antibody Cyclooxygenase-2, Leica Biosystems, Newcastle, United Kingdom). All tissue slides were stained and examined by the same expert pathologist.

COX-2 expression scoring

The scoring system described by Qiu et al. [12] was utilized. Specifically, each tissue sample was scored on a scale of 0 to 3 for COX-2 staining intensity (0=negative staining, 1=weakly positive, 2=moderately positive, 3=strongly positive) and on a scale of 0-4 for staining extent (0=negative, 1=1-25% of observed cells, 2=26-50% of cells, 3=51-75% of cells and 4=76-100%). The sum of both parameters was combined to derive the COX-2 expression score (on a scale of 0-7), with a score between 0 and 4 signifying low COX-2 expression and 5-7 high COX-2 expression, as previously described by Jeong et al. [13].

Statistical analysis

The assessment of the normality of data distribution was performed with the Shapiro-Wilk’s test. The Mann-Whitney-U (two-tailed) test was used to compare animal weight, COX-expression score, and TNFα serum levels between the UDCA and control groups. Pearson’s correlation was used to assess the relationship between COX-2 expression score and TNFα serum levels as well as Ki-67. Categorical data were compared with Fischer’s exact test. Data are presented as means ± standard deviation for categorical data and median and interquartile range (IQR) in the case of ordinal data. Results were considered statistically significant if the p-value was less than 0.05.

## Results

The mean weights of the animals in the UDCA group were 256.4±15.7 g and 263.6±14.8 g in the control group. A trend toward increased mitotic rates and Ki-67 was observed in the UDCA (43±11.4 and 13.7±24.7, respectively) compared to the control group (31±16.7 and 7.6±5.7, respectively, Table 1), albeit with no statistical significance (p=0.053 and 0.073, respectively).

COX-2 expression and staining

**Figure 1:**
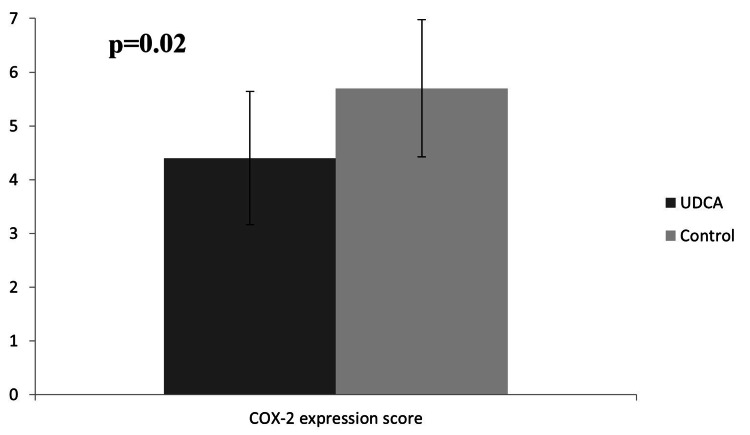
Comparative COX-2 expression score between the intervention and control groups.

**Figure 2:**
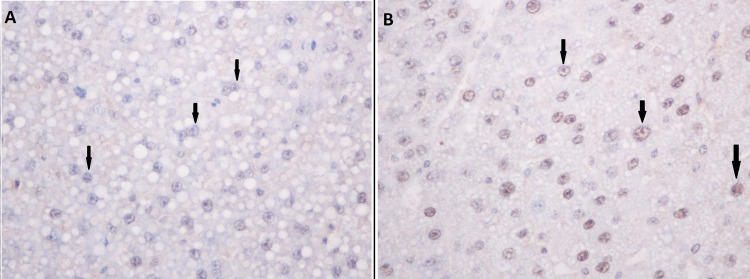
Immunohistochemical staining for cytoplasmic (black arrows) COX-2 in the UDCA (A) and control (B) groups. In both cases, a maximum COX-2 expression score of 7 was observed.

**Figure 3:**
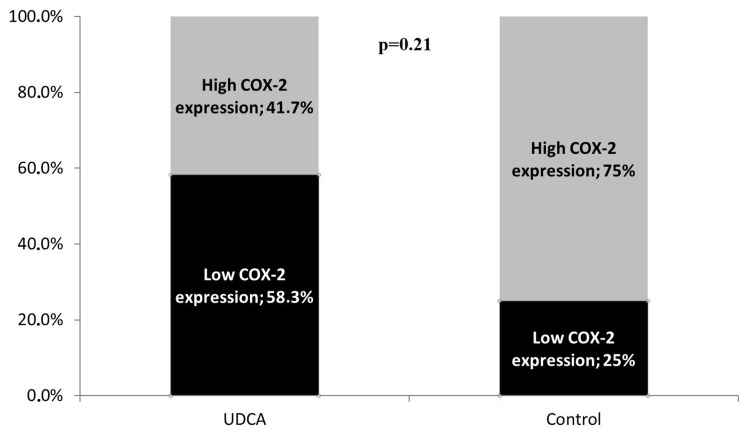
High COX-2 expression in the intervention and control groups. High COX-2 expression is defined as a score of 5 to 7.

Cytoplasmic hepatocyte COX-2 staining extent was significantly increased in the control group compared to the UDCA group (p=0.013, Table 1). The COX-2 staining intensity score was also increased in the control group although without statistical significance. Similarly, the final score of COX-2 expression was found to be increased in the control group (median value of 6 versus 4 in the UDCA group, p=0.028, Figure 1). In Figure 2, panels A and B demonstrate sampled tissue slides with a maximum COX-2 expression score of 7, belonging to the UDCA and control groups, respectively. The percentage of regenerating liver tissue samples with high COX-2 expression was higher in the control group versus the UDCA group (75% vs 41.7%, respectively, p=0.21, Figure 3).

TNFα measurements and correlations

**Figure 4:**
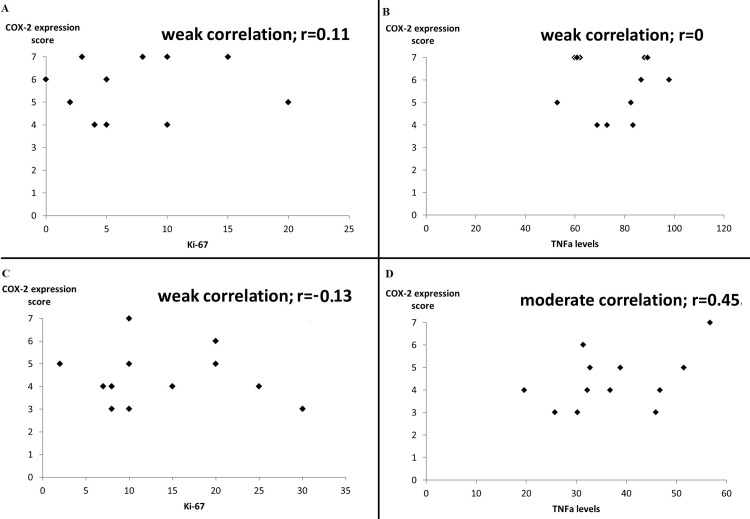
Correlation between COX-2 expression score and Ki-67 (A) or TNFα serum levels (B) in the control and UDCA (panels C and D, respectively) groups. The correlation coefficient r is expressed with values between −1 and 1.

Serum TNFα levels were found to be significantly increased in the control group (75.4±14.4 pg/mL) versus the UDCA group (37.3±10.9 pg/mL, p=0.004, Table 1). Specifically for the UDCA pretreated animal group, a moderate positive correlation (r=0.45) between COX-2 expression and TNFα levels was registered, while COX-2 expression and Ki-67 were only weakly correlated (r=−0.12). In the control group, similar comparisons revealed weak correlations for both parameters (r=0 and 0.11, respectively, Figure 4).

## Discussion

The present study is, to the best of our knowledge, the first report to verify the suppressive effect of UDCA on hepatic COX-2 expression. Expression of the isozyme was evidently lower in the pretreated group of animals (median score of 4 versus 6 in the UDCA group, p=0.028) with an observed 33.3% overall reduction in the percentage of specimens with high COX-2 expression (Figure 2).

UDCA has long been thought to enhance hepatocellular regeneration [6,14]. Khare et al. [9] were the first to observe that orally supplemented UDCA downregulated COX-2 expression in tumor colonocytes by reducing both deoxycholic acid-linked C/EPBbeta upregulation and COX-2 promoter activation. In turn, Liu et al. [15] demonstrated that suppressed C/EPBbeta activity led to a reduction in the interleukin-dependent activity of transmembrane and ubiquitin-like domain-containing protein 1 (Tmub1), an important intracellular controller of liver regeneration participating in a molecular cascade that incorporates STAT3 signaling [16]. It can therefore be surmised that the liver-protective effects of UDCA are attributable to inflammatory attenuation both on a systematic (as is evident by the reduction in circulating interleukin levels) and a local level [8,17]. On the other hand, complete blocking of the COX-2 enzyme proves detrimental for the regenerative process by creating prostaglandin E2 (PGE2) deficiency [5]. PGE2 is implicated to play a crucial role during the initial steps of the liver regeneration cascade with its levels increasing biphasically [18]. COX-2 targeting during the second peak of PGE2 synthesis arguably explains the net hepatoprotective effect of UDCA, in spite of reduced prostaglandin synthesis.

The experimental model of extended (80%) hepatectomy was carefully selected based on a growing body of evidence supporting that extreme resections entailing the removal of up to 90% of mouse liver parenchyma yield reproducible results with acceptable rates of animal survival [19,20]. This model aims to maximize the regenerative process and associated inflammatory cascade by inducing a setting of acute liver failure, compounded by a transient small-for-size liver syndrome [8]. Expression of COX-2 in liver tissue following resection is maximal 16 hours following the procedure, with its levels stabilizing after 96 hours, hence explaining our choice of terminating the experiment at 48 hours [5].

Increased circulating levels of TNFα coincide with increased hepatocellular expression of COX-2 regardless of the type of preceding liver injury [21]. Nuclear factor-kappa beta (NF-kB) is an important molecular mediator of the pro-inflammatory effects associated with TNFα by enhancing intracellular STAT3 signaling eventually leading to COX-2 mRNA induction [22,23]. Consequently, tissue COX-2 and serum TNFα levels increase concomitantly following extended hepatectomy and should be considered as manifestations of the same pro-inflammatory phenomenon that accompanies liver regeneration rather than independent phenomena [8]. Nevertheless, as far as the results of the present study are concerned, the levels of hepatic tissue COX-2 were unassociated with TNFα levels in the control group (r=0, Figure 4). Interestingly, pretreatment with UDCA appears to exert a potentiating regulatory effect on COX-2 expression, as is denoted by the stronger positive correlation observed in the UDCA-group animals (r=0.45, Figure 4). This particular finding suggests that UDCA acts by maximizing the effects of circulating proinflammatory cytokines (especially TNFα) on the regenerating liver tissue, possibly through molecular positive feedback loops mediated by NF-kB [24], while concurrently dampening the systematic component of the post hepatectomy inflammatory response as is demonstrated by the lower postoperative TNFα serum levels in UDCA pretreated animals. Conversely, Ki-67 expression was not found to be correlated with COX-2 expression in any of the animal groups (Figure 4), although Ki-67 expression was noted to be elevated in the UDCA group. These latter findings, when taken together, imply that although UDCA enhances liver regeneration, as previous studies on the topic demonstrate [6,7], its suppressive effect on COX-2 expression is, likely, of secondary importance.

The present study has several limitations. First, the study sample was estimated in order to evaluate differences in the expression of inflammatory markers (namely COX-2 and TNFα). It is, thus, possible that the study was underpowered to detect differences in other parameters such as Ki-67 expression and mitotic index. Moreover, although the obtained results unequivocally suggest that UDCA reduces COX-2 induction during the initiation of liver regeneration, the role of COX-2 during liver regeneration can only be indirectly assessed. Further elucidation of the molecular interplay mechanisms between UDCA and COX-2 is still required. Ultimately, the study results presented herein strongly indicate that the model of extensive 80% hepatectomy is appropriate to investigate the interaction of systemic inflammatory cascades with the regenerating liver parenchyma with inflammatory changes settling in liver tissue as early as 48 hours postoperatively.

## Conclusions

In conclusion, the present study demonstrates that preoperative administration of UDCA exerts a suppressive effect on tissue expression of COX-2 following 80% hepatectomy. In addition, UDCA enforces a positive correlation between COX-2 and serum TNFα levels, an effect that was not observed in the placebo-treated group of animals, suggesting that UDCA preconditions liver tissue to display an enhanced regenerative response to circulating cytokines, most notably TNFα. Finally, the weak association of COX-2 with Ki-67 expression levels suggests that COX-2 may be of secondary importance during the early phases of liver regeneration.
